# Treatment strategies for rituximab-resistant primary membranous nephropathy: from resistance mechanisms to emerging therapies

**DOI:** 10.3389/fimmu.2026.1844062

**Published:** 2026-06-11

**Authors:** Jingjie Gao, Yanan Pei, Lijuan Wei, Bo Yang, Hongtao Yang

**Affiliations:** 1 Department of Nephrology, First Teaching Hospital of Tianjin University of Traditional Chinese Medicine, Tianjin, China; 2 Department of Nephrology, National Clinical Research Center for Chinese Medicine, Tianjin, China

**Keywords:** B-cell-targeted therapy, complement inhibitors, precision immunotherapy, primary membranous nephropathy, rituximab resistance

## Abstract

With the expanding identification of target antigens in primary membranous nephropathy (PMN), treatment is shifting from empirical immunosuppression toward mechanism-based precision immunotherapy. Although rituximab (RTX) has substantially improved the management of PMN, a considerable proportion of patients still experience suboptimal response, relapse, or resistance. Accumulating evidence indicates that RTX resistance is a multifactorial process involving anti-drug antibody formation, reduced bioavailability, incomplete depletion of pathogenic B cells within lymphoid compartments, CD20 internalization and degradation, epitope spreading, persistence of autoantibodies against intracellular antigens, and genetic susceptibility. In response, a broad range of mechanism-guided therapeutic strategies is emerging, including next-generation anti-CD20 monoclonal antibodies, agents targeting distinct stages of B-cell differentiation, and advanced immune-engineering approaches such as CAR-T, CAAR-T, CAAR-NK, CAR-Treg, CAR-macrophage therapies, sweeping antibodies, antibody–drug conjugates, and bispecific autoantigen–T-cell engagers. In parallel, interventions targeting aberrant T–B cell crosstalk and complement activation are providing additional therapeutic opportunities for refractory disease. This review systematically summarizes the major pathogenic mechanisms underlying RTX-resistant PMN and integrates the latest advances in mechanism-based therapeutic strategies, with the aim of informing individualized treatment approaches and future translational research for refractory PMN.

## 1 Introduction

Primary membranous nephropathy (PMN) is one of the leading causes of nephrotic syndrome in adults. Its cardinal pathological feature is aberrant deposition of immune complexes on the epithelial side of the glomerular basement membrane (GBM), with immunofluorescence examination of renal biopsy specimens typically demonstrating granular deposits of immunoglobulin G (IgG) and complement component 3 (C3) (1). Traditionally, MN has been classified into two major subtypes based on the presence or absence of identifiable secondary causes: PMN and secondary membranous nephropathy (SMN). PMN accounts for approximately 70% of all MN cases, whereas SMN, which constitutes roughly 30% of cases, is frequently associated with autoimmune disorders, chronic infections, and malignancies (1–3). However, with breakthroughs in technologies for identifying PMN-associated target antigens, currently discovered target antigens cover 80–90% of patients with PMN. This research advancement renders the traditional “primary-secondary” dichotomous classification system inadequate for meeting the demands of contemporary etiological precision research and personalized clinical diagnosis and treatment. The reason lies in the distinct clinical phenotypes, pathological features, and disease prognoses associated with PMNs linked to different target antigens. Consequently, a novel classification framework centered on target antigen identification and integrated with disease-associated factors is being progressively proposed. Its core objective is to advance PMN diagnosis and treatment toward precision medicine (4). In recent years, Andeen and colleagues further proposed that the relationship between target antigens and disease is not strictly one-to-one. In clinical practice, clinical evaluation should not rely solely on the detection of target antigens; instead, targeted investigation of patients’ environmental exposure history and underlying comorbidities is essential, with the core objective being the identification of reversible causative factors (5). The perspective not only aligns with the trend toward precision medicine in modern healthcare but also places higher demands on optimizing and innovating PMN treatment strategies.

**Figure 1:**
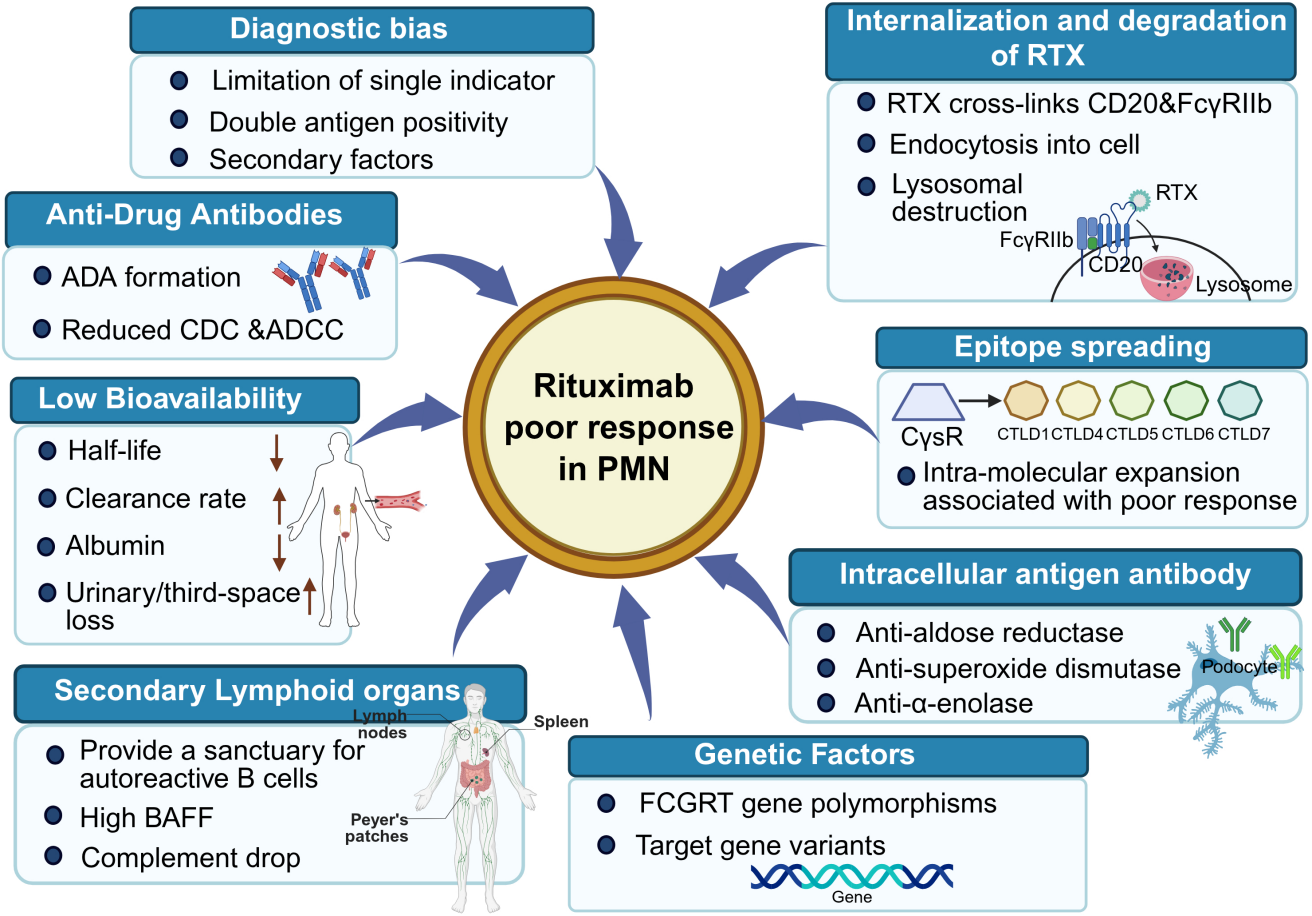
Poor response to RTX in PMN is multifactorial. Proposed mechanisms include anti-drug antibody formation, low bioavailability related to shortened half-life, incomplete depletion of autoreactive B cells in secondary lymphoid organs, rituximab internalization and lysosomal degradation, epitope spreading, the presence of autoantibodies against intracellular antigens, genetic susceptibility and diagnostic bias. Together, these factors may reduce B-cell depletion efficacy and contribute to treatment resistance or suboptimal response.

In tandem with the evolution of classification systems, treatment strategies for PMN are shifting from traditional broad-spectrum immunosuppression to targeted interventions at key pathogenic pathways. Currently, the optimal treatment strategy for PMN remains controversial. Available therapeutic agents include glucocorticoids, alkylating agents, calcineurin inhibitors, and biologic agents such as rituximab (RTX). Among these, the combination of cyclophosphamide and glucocorticoids remains one of the most extensively employed immunosuppressive protocols in current clinical practice, demonstrating definitive clinical efficacy in patients with moderate-to-high-risk PMN (6, 7). Its safety risks are primarily associated with cumulative dose, treatment duration, and the patient’s underlying individual risk profile, rather than representing an inherent limitation of the drug itself. Cyclophosphamide-related adverse events are more commonly observed in the context of higher cumulative exposure or repeated treatment courses (8, 9). Additionally, calcineurin inhibitors (such as tacrolimus and cyclosporine) are widely used in the treatment of PMN due to their specific inhibitory effect on T-cell activation. They are particularly suitable for patients who cannot tolerate cyclophosphamide therapy. Its main limitations lie in the relatively high risk of relapse after treatment discontinuation and the nephrotoxicity associated with long-term exposure (10, 11). These risks are closely related to treatment duration, cumulative drug exposure, baseline renal function, and the tapering or discontinuation strategy employed. RTX, as an anti-CD20 monoclonal antibody, offers advantages in terms of strong target specificity and a relatively lower incidence of adverse reactions, and has been recommended as a first-line therapeutic option for patients with PMN without significant comorbidities by multiple international clinical guidelines (8). However, it is worth noting that the response rate to RTX treatment has not reached an ideal level. Relevant studies report that approximately 35% of patients with PMN do not respond to RTX treatment, and the disease recurrence rate after treatment is as high as 27% (12, 13). Therefore, for this group of drug-resistant or relapsed patients, in-depth analysis of their resistance mechanisms is crucial for developing personalized treatment strategies. In this review, we summarize the current understanding of the mechanisms underlying RTX resistance in PMN (as shown in **Figure 1**) and discuss emerging mechanism-guided therapeutic strategies that may help overcome treatment resistance.

## 2 Mechanisms of RTX treatment failure

### 2.1 Development of anti-drug antibodies

One of the key mechanisms underlying RTX treatment failure is the development of anti-drug antibodies (ADAs) in a subset of patients (14). Previous studies have demonstrated that ADAs are detected in approximately 23%–43% of patients with PMN following RTX therapy (15, 16). Approximately 80% of ADAs have neutralizing activity against RTX (16), thereby reducing RTX-mediated complement-dependent cytotoxicity (CDC) and antibody-dependent cell-mediated cytotoxicity (ADCC), and significantly diminishing drug efficacy. In addition, ADAs can interfere with immunodiagnostic techniques, leading to false-positive test results and consequently affecting clinical judgment and treatment decisions (17). The clinical consequences of ADA development are multifaceted. By neutralizing RTX activity, depletion of CD20^+^ B cells becomes incomplete, and residual B cells may continue to proliferate and differentiate, ultimately leading to accelerated B-cell reconstitution and higher relapse rates (16). Additionally, the development of ADAs has also been demonstrated to be closely associated with patient prognosis. Boyer-Sauvet et al. report that patients with detectable ADAs exhibited more rapid B-cell recovery at 6 months and higher urinary protein levels at 12 months (16), indicating a clear association between positive ADA status and unfavorable renal outcomes. The generation of ADAs is not attributable to a single factor, but rather the combined result of the intrinsic immunogenicity of the drug, the intrinsic immune dysregulation of the disease, and the patient-specific metabolic status. As a chimeric mouse-human monoclonal antibody, RTX retains mouse-derived protein sequences with strong immunogenicity, which can be recognized as foreign by the immune system. Meanwhile, PMN itself is an autoimmune disorder characterized by defects in immune tolerance. This manifests as elevated anti-phospholipase A2 receptor (PLA2R) antibody levels, abnormal increases in interleukin (IL)-17 secretion, and PLA2R1 antigen epitope spreading (15). These immune abnormalities may predispose patients to ADA formation. Furthermore, vitamin D deficiency, particularly low 25-hydroxyvitamin D levels, is common in nephrotic syndrome. As a crucial immunomodulatory factor, insufficient levels of vitamin D can further disrupt immune homeostasis and increase the risk of developing ADAs (18). Currently, clinical management strategies for ADAs mainly involve two core approaches: ADA monitoring and substitution with humanized therapeutic agents. Given that ADAs typically develop progressively following RTX infusion and reach peak levels between months 9 and 12 (19), standardized ADA detection is recommended at 9 and 12 months post-infusion to minimize the risk of false-negative results. For patients planned to receive additional RTX therapy, ADA monitoring prior to re-infusion can help circumvent ineffective treatment and reduce unnecessary consumption of medical resources (14, 19). For patients with positive ADAs or refractory relapsing PMN, switching to humanized or fully human CD20 monoclonal antibodies (mAbs) (obinutuzumab, ofatumumab) may help overcome RTX resistance related to immunogenicity (20). Although these agents exhibit reduced immunogenicity and demonstrate characteristics analogous to endogenous human IgG, they cannot completely eliminate the immunogenicity of mAbs or prevent ADA formation (21). Currently, reliable methods for identifying individuals susceptible to ADA development remain lacking. Notably, studies in Crohn’s disease have suggested that polymorphisms in human leukocyte antigen (HLA) genes may be associated with ADA formation (22, 23). Therefore, future investigations are warranted to explore whether specific HLA alleles are associated with ADA generation in PMN, thereby establishing a biomarker system for predicting drug immunogenicity and providing a foundation for personalized therapeutic strategies.

### 2.2 Reduced bioavailability of RTX

Studies have demonstrated that patients with PMN exhibit significant pharmacokinetic abnormalities during RTX therapy compared with patients with other autoimmune diseases, including a shorter RTX half-life (24), higher clearance rates (25–27), and reduced RTX residual levels at 3 months post-treatment (28), indicating diminished *in vivo* bioavailability. The reduced bioavailability of RTX is likely multifactorial. Primarily, RTX distribution and action within the body depend on its binding to serum albumin. However, patients with PMN often exhibit impaired glomerular filtration barriers, leading to significant proteinuria and secondary hypoalbuminemia. This condition directly diminishes RTX’s ability to bind to its target proteins. Clinical data indicate that patients with baseline serum albumin levels below 22.5 g/L have an 8.66-fold increased risk of serum RTX levels falling to undetectable levels by month 3 of treatment compared to patients with normal albumin levels (29). Reduced albumin levels not only decrease the binding sites for RTX in the body but also accelerate drug clearance, resulting in a shorter half-life and diminished therapeutic efficacy. Additionally, RTX can be excreted in large quantities via the kidneys in urine. Previous studies have demonstrated that compared to individuals without proteinuria, patients with kidney disease exhibit a shortened duration of RTX exposure due to increased excretion of RTX in urine (28). Among these patients, those with proteinuria exceeding 8 g/day exhibited a significantly reduced clinical remission rate, a phenomenon attributed to the loss of RTX due to severe non-selective proteinuria (30). A similar phenomenon was observed in PMN: lower proteinuria selectivity was associated with reduced systemic exposure to RTX (31), resulting in poorer efficacy. Beyond urinary excretion, RTX can also be lost through the third space. In patients undergoing peritoneal dialysis, RTX may also be excreted via ascitic fluid. Relevant case reports indicate that RTX concentrations in ascites fluid from such patients can reach up to 3518 μg/L. Furthermore, excretion levels correlate positively with IgG concentrations in ascites fluid, suggesting that third-space loss represents a significant alternative pathway for drug depletion beyond urinary excretion, thereby further exacerbating inadequate drug exposure (32). Several studies have demonstrated an association between insufficient serum RTX concentrations and reduced therapeutic efficacy in patients with PMN. Clinical observational data from patients with PMN demonstrated that approximately 56% of patients had undetectable effective concentrations of RTX in serum at 3 months post-administration. These patients exhibited lower clinical remission rates at 6 and 12 months and prolonged time to achieve clinical remission. Further analysis revealed that inadequate drug exposure was correlated with markers of disease severity, including higher baseline PLA2R1 titers and lower serum albumin levels (29). Additionally, the research team has developed a detection tool capable of quantifying RTX levels in urine. This tool facilitates precise assessment of drug loss and holds promise as an adjunct for guiding individualized treatment in patients with PMN (31).

For patients receiving RTX therapy, individualized treatment adjustments may be required based on immunological monitoring. For patients with undetectable serum RTX levels at 3 months who remain in active disease status, additional RTX infusions may be planned with optimized supportive care to achieve higher serum RTX concentrations, thereby increasing the likelihood of remission and shortening the duration of active disease. Supplemental RTX dosing should be considered within 3 months. For patients with PMN with severe non-selective proteinuria at baseline, appropriately increased RTX doses may be administered on top of standard dosing, or high-dose regimens may be considered to compensate for drug loss in urine. Regular monitoring of serum RTX levels, urinary RTX concentrations, and anti-PLA2R antibody titers, together with assessment of renal function and proteinuria, may help dynamically evaluate drug exposure and disease activity and facilitate timely adjustment of treatment strategies.

### 2.3 Incomplete clonal expansion of self-reactive B cells in lymphoid organs

Although RTX effectively depletes B cells in peripheral blood, its depletion efficacy is significantly attenuated in secondary lymphoid organs (SLO) such as lymph nodes (33), and this tissue-selective limitation represents an important mechanism contributing to PMN relapse. Incomplete depletion of autoreactive B cells within SLOs allows these tissues to act as reservoirs for residual B-cell activation, antibody production, and T-B cell interactions. These residual B cells can continuously differentiate into plasma cells, perpetuating the production of pathogenic antibodies and thereby triggering PMN relapse (34). Additionally, RTX therapy may also induce phenotypic remodeling of B cells within lymph nodes, characterized by a reduction in naive B-cells and a marked increase in memory B-cell proportions. Memory B cells, which express high levels of survival-related molecules, can sustain aberrant immune responses (35), serving as an immunological reservoir for PMN relapse. Concurrently, the unique microenvironmental characteristics of SLO further attenuate the cytotoxic effects of RTX, exacerbating the risks of drug resistance and relapse. On one hand, elevated B-cell activating factor (BAFF) levels in SLO promote B-cell survival, partially counteracting the B-cell-depleting effects of RTX. On the other hand, local complement exhaustion diminishes RTX-dependent CDC efficacy, thereby reducing the cytotoxicity against B cells (36, 37). The spleen, as a key lymphoid organ, may also serve as a sanctuary for RTX-resistant autoreactive memory B cells and long-lived plasma cells. These cells are capable of surviving RTX therapy and may become reactivated after B-cell reconstitution. Upon reactivation, they can differentiate into antibody-producing plasma cells and initiate germinal center (GC) reactions, ultimately driving pathogenic antibody production and establishing a cycle of disease relapse (38).

### 2.4 RTX internalization and degradation in B cells

Antibody internalization represents a key mechanism contributing to reduced therapeutic efficacy in PMN treatment. The process begins with the binding of RTX to CD20 molecules on the B-cell surface, followed by cross-linking with Fc gamma receptor IIb (FcγRIIb) expressed on the same B cell to form a trimolecular complex. Upon formation of this complex, phosphorylation of the intracellular domain of FcγRIIb is triggered, leading to internalization of the complex and its subsequent lysosomal degradation (39). This process shortens the half-life of RTX *in vivo* and attenuates its effector functions. The rate of antibody internalization is negatively correlated with B-cell depletion efficiency, and this characteristic is observed across different types of anti-CD20 mAbs. As a type I anti-CD20 monoclonal antibody, RTX exhibits a relatively rapid internalization rate, which consequently results in relatively limited B cell depletion efficiency. In contrast, type II anti-CD20 mAbs display slow internalization rates, allowing prolonged retention on the B-cell surface and maintaining stronger targeted cytotoxic activity, thereby achieving superior B-cell depletion efficacy (40), but the specific mechanisms underlying this process remain incompletely understood in the context of PMN. Given the impact of internalization mechanisms on therapeutic efficacy, clinicians may consider the use of type II CD20 mAbs with lower internalization rates, such as obinutuzumab, as an alternative. This approach may reduce poor response or relapse to RTX therapy antibody internalization, enhances response rates, and offers a new treatment option for patients with poor response or relapse to RTX therapy.

### 2.5 Autoantigen epitope spreading

During the progression of an immune response, antigen recognition may extend from the initial dominant epitopes to additional secondary epitopes, a phenomenon known as epitope spreading (41). Epitope spreading can be broadly classified into intramolecular and intermolecular epitope spreading (42). Intramolecular epitope spreading refers to the expansion of immune responses from one epitope to other epitopes within the same antigen molecule (43), whereas intermolecular spreading involves immune responses directed against epitopes from different antigen molecules that may originate from the same tissue or from distinct tissues (44). Epitope spreading represents a double-edged sword: while it can enhance immune defense against pathogens, it may also amplify immune responses against self-antigens in autoimmune diseases, thereby promoting disease progression (45). Intramolecular epitope spreading has been well documented in PMN. In the Heymann nephritis rat model, experimental studies have demonstrated that epitope spreading occurs during disease progression and is associated with increased disease severity, particularly with worsening proteinuria (46). In PLA2R-associated MN, the PLA2R molecule comprises multiple domains including the N-terminal cysteine-rich domain (CysR), fibronectin type II-like domain, and eight C-type lectin-like domains (CTLDs). Among these, CysR is considered the immunodominant epitope. As the disease progresses, immune recognition may extend from CysR to other domains such as CTLD1 and CTLD7, resulting in a multi-epitope recognition pattern that is associated with higher disease activity (47). Compared with intramolecular spreading, direct evidence for intermolecular epitope spreading in PMN remains limited; however, several clinical observations support its potential existence. For instance, dual antigen positivity has been reported in some patients (48–51), and the coexistence of antibodies against different antigens in MN complicated by autoimmune thyroiditis suggests that immune responses may cross-react across distinct antigen molecules (52). Recently, Liu et al. employed yeast surface display technology to identify novel epitope regions in PMN, such as CTLD4, CTLD5, and CTLD6, further confirming the prevalence of epitope spreading in patients with PMN (53).

Whether epitope spreading can serve as a biomarker for predicting adverse renal outcomes and monitoring disease prognosis remains currently debated (54, 55). Some studies suggest that the prognostic value of epitope spreading may depend largely on total antibody titers, with adverse outcomes in patients with high antibody levels possibly reflecting the overall antibody burden rather than epitope spreading itself (56). Nevertheless, accumulating evidence indicates that epitope spreading may predict clinical remission and disease prognosis in patients with PMN (53, 57, 58). A recent retrospective study evaluating the predictive value of PLA2R epitope spreading for proteinuria following RTX therapy found that PLA2R epitope spreading was an independent risk factor for persistent proteinuria six months after treatment (59). The concept of epitope spreading also provides a rationale for stratified therapeutic strategies in PMN. For patients with restricted epitope responses targeting solely the CysR domain, a period of observation for approximately six months followed by reassessment may be appropriate. In contrast, patients exhibiting epitope spreading may benefit from earlier intervention with intensified RTX regimens. For patients who respond poorly to RTX, next-generation anti-CD20 monoclonal antibodies may be considered, as their stronger B-cell depletion capacity may more effectively suppress multi-epitope immune responses (60).

### 2.6 Aberrant expression of intracellular antigen autoantibodies

Studies have confirmed that autoantibodies targeting various intracellular podocyte antigens can be detected in a substantial proportion of patients with PMN, including antibodies against aldose reductase, superoxide dismutase 2 (SOD2), and α-enolase (αENO) (61–64). Cohort studies indicate that the presence of circulating anti-SOD2 and anti-αENO autoantibodies is independently associated with poor outcomes, including poor proteinuria resolution and decline in estimated glomerular filtration rate (eGFR). Multivariate analyses further demonstrated that positivity for intracellular autoantibodies provides additional prognostic value in predicting adverse clinical outcomes. Patients with high titers of antibodies against PLA2R, SOD2, and αENO were associated with risks of unremitted proteinuria and eGFR decline at 12 months, with patients positive for all three antibodies exhibiting the highest risk of adverse outcomes, whereas PLA2R1/Thrombospondin Type-1 Domain-Containing Protein 7A (THSD7A) double-negative patients demonstrated the most favorable prognosis (65). Currently, the pathogenic mechanisms of intracellular antigens remain unclear. These autoantibodies may reflect a more complex immunological landscape in PMN. It has been proposed that the immunological process in PMN initiates with autoantibody responses against podocyte surface antigens, followed by disease progression with subsequent intermolecular or intramolecular spreading, thereby inducing the production of secondary autoantibodies targeting intracellular antigens (65). Intracellular antigens represent a more complex immunological spectrum, often predicting more refractory disease, slower treatment responses, and higher relapse susceptibility. Patients with such profiles may exhibit reduced sensitivity to single-agent anti-CD20 depletion regimens (e.g., RTX). In conclusion, as detection technologies continue to mature, centers with adequate capabilities may additionally monitor levels of intracellular antibodies such as SOD2 and αENO in refractory patients with PMN to dynamically assess disease progression and therapeutic response.

### 2.7 Genetic polymorphisms

The neonatal Fc receptor (FcRn) represents another critical factor influencing the efficacy of therapeutic mAbs such as RTX. Encoded by the FCGRT gene, polymorphisms in the variable number tandem repeat (VNTR) region of its promoter directly determine antibody retention efficiency and tissue distribution characteristics *in vivo*. Patients homozygous for the VNTR3/VNTR3 exhibit higher FcRn expression levels and significantly superior binding and recycling capacity for IgG-class antibodies compared with VNTR2/VNTR3 heterozygous patients, which facilitates increased antibody accumulation in target tissues and prolongs effective drug concentrations (66). This may help explain suboptimal therapeutic responses observed in certain patient populations. Additionally, genetic variations constitute an important factor influencing drug efficacy. For instance, a previous study identified that a single missense mutation in the gene encoding complement protein C5 resulted in poor response to eculizumab therapy (67). Genetic mutations can alter the structure of drug targets, thereby impairing antibody-target binding. Studies have revealed that multiple single-nucleotide variants (SNVs) were identified within the corresponding epitope regions for each analyzed antibody (68), with a substantial proportion of these SNVs predicted to alter antibody binding—either by disrupting the antibody-antigen interface without affecting antigen structure and function, or indirectly through modifying the antigen’s intrinsic conformation. In the clinical application of therapeutic antibodies such as anti-CD20 and anti-CD38 agents, drug resistance mediated by target gene variants has emerged as a well-defined clinical issue and provides guidance for treatment regimen selection. For instance, the CD20 N171Y mutation confers RTX resistance but does not affect obinutuzumab efficacy. Therefore, for carriers of the CD20 N171Y variant, clinical guidelines recommend prioritizing obinutuzumab over RTX to circumvent resistance (69). This suggests that therapeutic failure in some patients may not reflect inherent drug inefficacy, but rather the presence of genetic variants in pathogenic targets. For example, PLA2R contains multiple epitopes, and if a critical epitope undergoes mutation, antibodies targeting that specific epitope will become ineffective. Different variants within the same target exhibit differential sensitivities to distinct antibodies. In PMN treatment, if a patient demonstrates resistance to a particular antibody, detection of target variants should be performed when feasible, and switching to an antibody targeting a different epitope may restore therapeutic efficacy. Future strategies involving pre-emptive sequencing of pathogenic target genes and FCGRT polymorphisms, combined with the epitope specificity of antibody therapeutics and development of multi-epitope agents, hold promise for addressing certain resistance mechanisms (68).

### 2.8 Initial diagnostic misclassification and disease heterogeneity

Poor response to RTX should not be automatically equated with true drug resistance; diagnostic misclassification, incomplete disease stratification, or unrecognized secondary causes often warrant higher priority consideration. Although anti-PLA2R antibodies are a key biomarker for PMN with high specificity, their sensitivity remains limited (70, 71), and discordance between circulating anti-PLA2R antibodies and glomerular PLA2R antigen expression is not uncommon (72, 73). Thus, reliance on a single biomarker is insufficient to fully capture the immunological heterogeneity of PMN. More importantly, PLA2R positivity does not unequivocally indicate PMN. While PLA2R was previously considered largely specific to PMN, accumulating evidence indicates that PLA2R positivity may also occur in lupus-associated, infection-related, malignancy-associated, and drug-induced MN, suggesting that secondary etiologies cannot be excluded solely on the basis of anti-PLA2R positivity (74, 75). The identification of novel target antigens has further reshaped the classification framework of MN. Some patients exhibit dual antigen positivity, and these individuals often experience delayed clinical remission compared with those with isolated PLA2R positivity, implying a more complex immunopathogenic background in which B-cell depletion alone may be insufficient (76). In parallel, specific antigen profiles may provide important etiological clues: NEL-like protein 1 and THSD7A are frequently associated with malignancy (77); exostosin 1/exostosin 2 (EXT1/EXT2) and neural cell adhesion molecule 1 are more commonly linked to lupus-associated MN (78, 79); neuron-derived neurotrophic factor has been associated with syphilis-related MN (80); contactin 1 (CNTN1) may coexist with chronic inflammatory demyelinating polyneuropathy (81), and protocadherin 7 has been implicated in drug-associated MN (82). Therefore, in patients with persistent proteinuria despite adequate peripheral B-cell depletion following standardized RTX therapy, indiscriminate escalation of immunosuppression should be avoided. Instead, the underlying disease biology should be reassessed. In this context, target antigen profiling, integrated with detailed clinical history, malignancy screening, and evaluation for autoimmune or infectious comorbidities, may offer substantially greater clinical value than simply switching to alternative immunosuppressive agents.

In summary, the multifactorial mechanisms underlying RTX resistance underscore the limitations of single-target B-cell depletion strategies. Reliance solely on CD20-targeted therapy is unlikely to completely interrupt the pathogenic pathways driving PMN or fully overcome therapeutic resistance. Therefore, the development of multi-pathway and multi-layered immunotherapeutic strategies will be essential for achieving more durable and comprehensive disease control.

## 3 B-cell-targeted drugs

**Figure 2:**
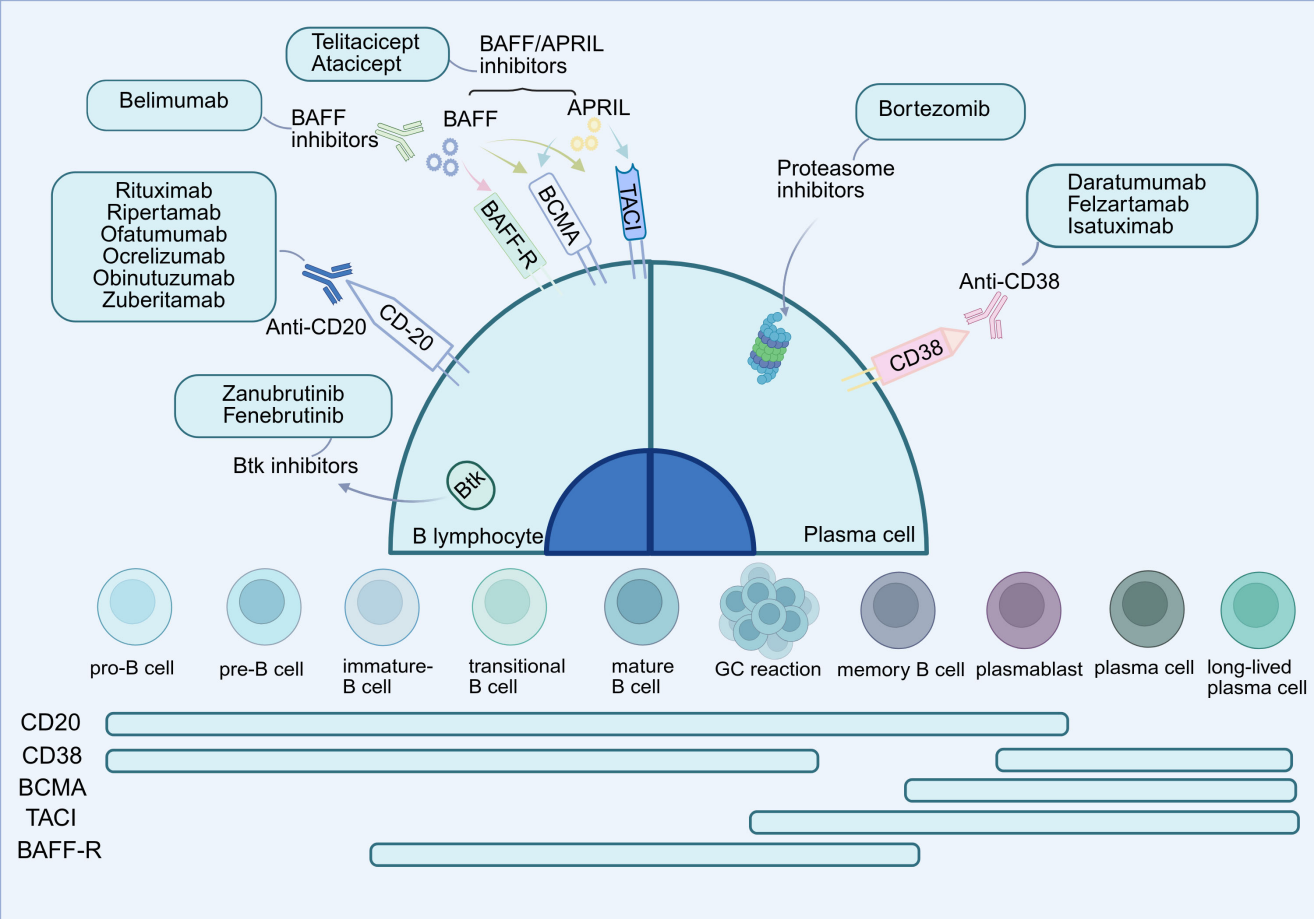
Stages of B-cell development and mechanisms of targeted therapeutic interventions: The differentiation of hematopoietic stem cells into mature B cells and plasma cells is a highly regulated, multistep process characterized by the dynamic expression of surface markers, including CD20, CD38, BCMA, TACI, and BAFF-R. These molecules serve as key therapeutic targets for B cell–directed interventions. Targeted therapies selectively eliminate or functionally modulate pathogenic B-cell populations by acting on distinct developmental stages or critical signaling pathways. This stage-specific targeting provides a mechanistic rationale for combination therapeutic strategies.

CD20 mAbs represent a core therapeutic approach targeting malignant B cells. Recent drug development efforts have focused on structural optimization and efficacy enhancement, offering more targeted alternatives to conventional regimens. Key advances include (as shown in **Figure 2**):

### 3.1 Novel anti-CD20 monoclonal antibodies

Ripertamab, an engineered derivative of RTX, has been modified within the constant heavy chain 1 domain to better match the prevalent G1m (1, 17) immunoglobulin allotype in Asian populations (83). Multicenter studies have demonstrated that ripertamab has efficacy comparable to that of RTX in terms of proteinuria reduction, serum albumin recovery, and decline in anti-PLA2R antibody titers in patients with MN. Importantly, ripertamab has been associated with lower rates of infusion reactions and adverse events at a more favorable cost (84), suggesting that it may represent a viable alternative for patients who are intolerant of or respond inadequately to RTX. Ofatumumab, a fully human monoclonal antibody, enhances CDC through the epitope proximity to the cell membrane (85–87). Subcutaneous administration enables targeting of B cells within lymph nodes, disrupting autoantibody production (88, 89). Clinical studies have reported favorable outcomes in patients with RTX-resistant PLA2R-associated PMN, with sustained remission and an acceptable safety profile. In addition, the pharmacokinetic characteristics of subcutaneous administration may allow outpatient or home-based treatment, which could potentially improve treatment adherence (90, 91). Reported adverse events are generally mild and manageable, with infusion-related reactions typically resolving after temporary treatment interruption (92). Additionally, after engineering optimization, the humanized mAbs obinutuzumab and ocrelizumab exhibit enhanced affinity for the Fc gamma receptor IIIa receptor on natural killer (NK) cells, amplifying ADCC to achieve deeper and more sustained B-cell depletion (86, 93). Among these, obinutuzumab demonstrates superior efficacy to RTX in PMN treatment and exhibits significant salvage therapeutic value for patients with suboptimal RTX responses, and is capable of inducing durable remission (94, 95). Currently, ocrelizumab has only been reported in isolated case studies to improve antibody levels and proteinuria in patients with multiple sclerosis complicated by PMN (96). Its broader clinical applicability requires confirmation through prospective clinical trials. Regarding safety profile, infusion-related reactions and respiratory tract infections constitute the primary concerns, though these can be mitigated through premedication, with overall favorable safety demonstrated (97, 98).

### 3.2 BAFF inhibitors

In addition to antibodies that directly target CD20, inhibitors of B-cell survival factors, such as BAFF, also offer new therapeutic avenues for PMN treatment. Belimumab, a humanized IgG1-kappa (IgG1-κ) monoclonal antibody, specifically binds soluble BAFF trimers, blocking their interaction with receptors and thereby inhibiting B-cell activation and proliferation. Unlike RTX, belimumab exerts broader regulatory effects on B-cell biology by blocking naive B-cell maturation, suppressing memory B-cell self-renewal, and inhibiting the differentiation of short-lived plasma cells (SLPCs), thereby reducing autoantibody production (99–101). Clinical studies demonstrated that among 14 patients with persistent PMN receiving belimumab therapy, nine achieved complete or partial remission, with significant reductions in both anti-PLA2R antibody levels and proteinuria; patients with abnormal baseline albumin and cholesterol levels also normalized at final follow-up. No severe adverse events occurred during treatment, indicating favorable safety profiles (102). The ongoing multicenter clinical trial (NCT03949855) is further investigating its efficacy in combination with RTX, aiming to enhance remission rates in refractory patients through dual targeting. This combination is supported by a clear theoretical basis: following RTX treatment, serum BAFF levels increase due to negative feedback regulation, which may accelerate B-cell reconstitution and contribute to disease relapse. Belimumab can neutralize elevated BAFF, prolonging the duration of B-cell depletion (103). It should be noted that belimumab only recognizes soluble BAFF trimers and cannot bind to the more potent 60-mer structure, potentially limiting its efficacy during periods of high disease activity (99). Furthermore, it acts exclusively on SLPCs and cannot eliminate long-lived plasma cells (LLPCs), which are the primary source of persistently high-titer anti-PLA2R antibodies (104). Therefore, monotherapy is more suitable for patients in the early stages of disease, where the LLPC pool has not yet been established and antibody titers are moderate to low. The efficacy, safety, and optimal patient selection for combination strategies require further validation in large-scale clinical studies.

### 3.3 Dual inhibition of BAFF/APRIL

To overcome the limitations of single-agent BAFF inhibition, dual inhibitors targeting both BAFF and a proliferation-inducing ligand (APRIL) have emerged. These agents simultaneously block two key survival signals for B cells and, through APRIL inhibition, can also affect long-lived plasma cells (LLPCs) and reducing antibody production from this source (105), thereby compensating for the deficiencies of single-target therapeutics. The representative agent atacicept is a fusion protein composed of the extracellular domain of the transmembrane activator and CAML interactor (TACI) linked to the Fc fragment of IgG. Early clinical development was temporarily halted due to its high affinity for APRIL, which led to over-suppression of LLPCs, potentially causing severe infections and hypogammaglobulinemia (106, 107). Recent studies demonstrate its efficacy in IgA nephropathy, suggesting atacicept retains therapeutic potential for BAFF/APRIL-related disorders (108). Telitacicept is a structurally optimized fusion protein that, in addition to modulating B-cell responses, may indirectly influence T-cell helper function through TACI-related mechanisms, thereby exerting broader immunomodulatory effects (109–111). Additionally, through TACI expression on T cells, it modulates T-cell helper functions, thereby achieving more comprehensive immunomodulation (112, 113). Although large-scale randomized controlled data are currently lacking, existing case reports have demonstrated its potential value in specific patient populations: MN patients who are positive for anti-PLA2R antibodies and intolerant to glucocorticoids, as well as those with poor response to RTX therapy, have achieved varying degrees of clinical remission following treatment with telitacicept as monotherapy or in combination therapy (111, 114). Telitacicept primarily suppresses aberrant B-cell immunity through sustained modulation of the BAFF/APRIL axis, rather than inducing rapid cellular depletion. In contrast, CD20 mAbs rapidly eliminate peripheral B cells but are ineffective against CD20-negative plasma cells and tissue-resident B cells (115). Based on this mechanistic complementarity, a sequential strategy employing initial RTX induction followed by telitacicept maintenance has emerged as a cutting-edge therapeutic approach for refractory autoimmune diseases. Clinical studies demonstrated that this regimen achieves superior complete remission rates, greater reductions in proteinuria, and higher antibody seroconversion rates compared with monotherapy in patients with refractory lupus nephritis (LN) (116). Given that both PMN and LN share aberrant B-cell activation as their core pathological mechanism, this sequential strategy warrants future investigation in the PMN field, with the potential to enhance therapeutic efficacy in refractory cases through mechanistic synergy and provide novel therapeutic insights.

### 3.4 Protease inhibitors

For refractory PMN characterized by persistent antibody-driven disease activity, directly targeting antibody-secreting cells has emerged as another important therapeutic strategy. Bortezomib, a reversible proteasome inhibitor, reduces inflammatory cytokines by blocking the nuclear factor kappa B pathway and induces plasma cell apoptosis through endoplasmic reticulum (ER) stress (117). In PMN, LLPCs bear an exceptionally high ER protein-folding burden due to continuous antibody synthesis, rendering them particularly sensitive to proteasome inhibition (118). Currently, the evidence for bortezomib in PMN is primarily derived from exploratory use in refractory patient populations. Case reports have demonstrated that bortezomib is effective in treating RTX-resistant PMN. Both monotherapy and combination therapy with dexamethasone have been reported to reduce autoantibody titers, decrease proteinuria, and stabilize renal function, thereby representing a potential salvage therapeutic option following RTX resistance (119–121). Regarding safety, given the limited clinical experience with bortezomib in PMN, its adverse event profile remains insufficiently characterized. In other clinical settings, the most commonly observed adverse effects include peripheral neuropathy and gastrointestinal symptoms. These toxicities are generally manageable, and short-term treatment is usually well tolerated (122).

### 3.5 CD38 monoclonal antibody

LLPCs predominantly exhibit a CD38-positive phenotype, and their sustained production of autoantibodies represents a critical factor in the chronic progression of PMN. Accordingly, CD38 has emerged as a promising therapeutic target for refractory PMN. Daratumumab is an IgG1-κ monoclonal antibody that induces target cell apoptosis through multiple mechanisms, including CDC, ADCC, antibody-dependent cellular phagocytosis and direct apoptotic signaling (69, 123, 124). Currently, isolated case reports in patients with relapsed or refractory PMN after failure of multiple lines of therapy have suggested its potential efficacy, providing preliminary evidence to support the application of daratumumab in PMN (125, 126). In addition to daratumumab, several novel anti-CD38 monoclonal antibodies have been developed. Isatuximab has a mechanism of action broadly comparable to that of daratumumab. However, studies in kidney disease remain lacking, and its application in PMN warrants further investigation (127). By contrast, the novel anti-CD38 monoclonal antibody felzartamab has advanced more rapidly in the clinical translation for PMN. Felzartamab has demonstrated promising efficacy in a clinical trial (NCT04145440), with most high-risk anti-PLA2R-positive MN patients achieving immunological remission. The treatment was generally well tolerated, exhibiting a low incidence of serious adverse events (128). Overall, anti-CD38 antibodies represent an emerging therapeutic strategy with strong mechanistic appeal, particularly for patients with RTX-refractory disease. However, the overall evidence remains at an early stage and is insufficient to support routine clinical use. Given the complementary target cell spectra of anti-CD20 and anti-CD38 therapies, combination or sequential treatment strategies warrant further investigation.

### 3.6 BTK inhibitors

Bruton’s tyrosine kinase (BTK) is a central regulator of B-cell maturation, activation, and signaling. Inhibition of BTK suppresses autoreactive B-cell activation by blocking downstream signaling of the BCR, thereby representing a promising therapeutic strategy for PMN (129). Early evidence supporting the autoimmune applications of the first-generation covalent BTK inhibitor ibrutinib has been primarily derived from animal models, particularly LN (130). Clinical evidence has likewise been largely limited to case reports involving patients with chronic lymphocytic leukemia–associated SMN (131). Meanwhile, its off-target inhibition of vascular endothelial growth factor receptor may induce glomerular endothelial injury (132). These toxicities have limited the routine use of first-generation inhibitors in patients with PMN and have driven the development of newer agents with greater selectivity. Among second-generation inhibitors, zanubrutinib exhibits higher selectivity and lower toxicity, making it more suitable for long-term maintenance therapy. Currently, phase II/III clinical trials (NCT05707377) are evaluating its efficacy in combination with tacrolimus for PMN treatment. Fenebrutinib, another second-generation agent, is a non-covalent, reversible BTK inhibitor. Unlike first-generation inhibitors, it does not rely on covalent binding to the Cys481 residue and therefore overcomes resistance associated with C481S mutations, maintaining potent inhibitory activity against mutant BTK (133). Additionally, fenebrutinib demonstrates high selectivity, reduced off-target effects (134), and favorable pharmacokinetic properties (135). In summary, by virtue of their unique molecular mechanism of interrupting activation signaling, BTK inhibitors offer a differentiated and complementary therapeutic avenue for drug development in PMN. However, their efficacy in PMN remains to be confirmed in larger prospective clinical trials.

## 4 Engineered chimeric antigen receptor (CAR) cell therapy

### 4.1 CAR-T cell therapy

CAR-T cell therapy represents a novel therapeutic paradigm for refractory PMN, with the key advantage of achieving deep and durable depletion of pathogenic B cells. Current CAR-T strategies in PMN are primarily focused on the selection of B cell surface markers and combinatorial design. The most commonly used B-cell marker is CD19. Its key advantage lies in the broad expression of CD19 across the B-cell lineage and its high antigen specificity, enabling more comprehensive elimination of pathogenic B cells (136, 137). A clinical trial for CD19 CAR-T therapy in PMN is currently recruiting participants (NCT06690359), with its efficacy and safety awaiting further validation. Given that a subset of pathogenic antibodies may originate from CD19-negative, BCMA-positive long-lived plasma cells (LLPCs) in the bone marrow, BCMA-targeted CAR-T therapy may serve as a critical complementary strategy for refractory patients (138). To further enhance depletion efficiency and prevent antigen escape, bispecific CAR-T cells simultaneously targeting CD19 and BCMA have entered the translational stage, and a related clinical trial in PMN (NCT06285279) is currently underway. In addition to classical targets such as CD19 and BCMA, emerging CAR designs may target the BAFF/BAFF-R signaling axis to modulate B-cell survival and activation. Moreover, BAFF-based strategies act at the level of the BAFF signaling axis, simultaneously influencing multiple B-cell subsets through BAFF-R, TACI, and BCMA. Therefore, BAFF-CAR represents a broader immunomodulatory approach rather than a redundant targeting strategy. This mechanism has been validated in animal models of lupus and holds promise for providing additional treatment options for patients with PMN in the future (139).

**Figure 3:**
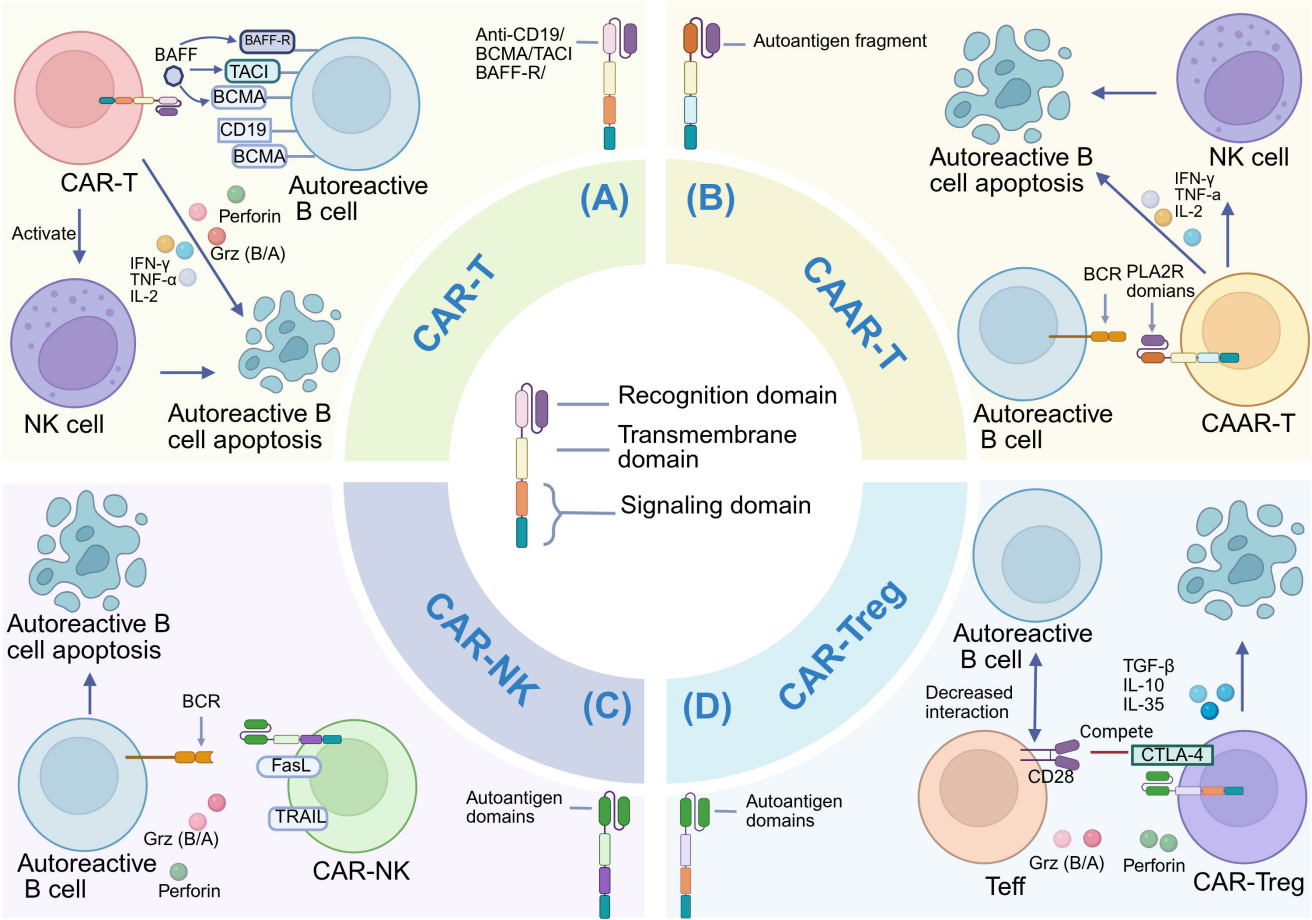
CARs expressed on CAR-T cells contain a single-chain variable fragment (scFv) that recognizes target antigens such as CD19 and BCMA on B cells. In addition, some CAR designs may target the BAFF–BAFFR signaling axis to modulate B-cell activation and survival, thereby enhancing the clearance of autoreactive B cells. In contrast, CAAR-T cells express a modified CAR in which the scFv is replaced by the extracellular domain of autoantigens (e.g., PLA2R), enabling specific recognition of autoreactive B cells via their antigen-specific BCRs. Upon antigen engagement, CAR/CAAR signaling induces T-cell activation, leading to direct cytotoxicity against autoreactive B cells primarily through the release of perforin and granzymes (Grz), along with cytokine secretion. These cells may also contribute to the activation of innate immune responses, including NK cells. CAAR-NK cells similarly recognize autoreactive B cells expressing BCRs specific for PLA2R or THSD7A, inducing target cell apoptosis via cytotoxic mediators such as perforin and Grz, as well as through death receptor pathways involving Fas ligand (FasL) and tumor necrosis factor-related apoptosis-inducing ligand (TRAIL). CAR-Treg cells suppress immune responses through multiple mechanisms, including the secretion of immunosuppressive cytokines (e.g., TGF-β, IL-10, and IL-35), inhibition of B-cell differentiation into plasma cells, and reduction of pathogenic antibody production. In addition, they compete with CD28 for binding to CD80/CD86 via CTLA-4, thereby disrupting T–B cell interactions and inhibiting GC formation. CAR-Tregs can also induce apoptosis of effector T cells through Grz-dependent mechanisms.

The mechanism of action of CAR-T cell therapy in PMN involves the binding of CAR-T cells (targeting CD19 or BCMA) to antigen-expressing cells, leading to T-cell activation, releasing cytotoxic molecules and cytokines to directly kill autoreactive B cells, while simultaneously recruiting other immune system components to assist in the clearance process (as shown in **Figure 3A**) (140). Additionally, CAR-T cells can persist long-term *in vivo*, establishing sustained immune surveillance and memory responses to effectively prevent disease relapse. Compared with conventional targeted agents, CAR-T cells exhibit superior tissue penetration, potentially achieving deeper B-cell depletion (141–143), thereby addressing the incomplete clearance of autoreactive B cells in lymph nodes or target organs observed with RTX and other targeted therapeutics. Unlike antibody drugs with specific half-lives, CAR-T cells can undergo sustained proliferation and long-term persistence within the body. This may enable sustained clearance of diseased cells, further enhancing the long-term efficacy of treatment (144). The specific risk factors associated with CAR-T therapy in patients with PMN have not yet been fully defined. Short-term adverse events may include acute cytokine release syndrome (CRS) and reversible neurotoxicity. Potential long-term consequences include persistent B-cell depletion, impaired B-cell reconstitution, and hypogammaglobulinemia, thereby substantially increasing the risk of infection. Its safety and efficacy still require validation in large-scale clinical studies (145). To address these limitations, transiently expressed mRNA CAR-T cell therapy has been proposed (146, 147), which can effectively reduce toxicity risks associated with long-term persistence of conventional CAR-T cells, providing a novel direction for optimizing CAR-T therapeutic strategies in PMN. Further investigation of its therapeutic potential may offer support for overcoming bottlenecks in traditional CAR-T therapy.

### 4.2 Chimeric autoantibody receptor T (CAAR-T) cell therapy

CAAR-T is an engineered variant of CAR-T cell therapy that represents a more precise antigen-specific immunotherapeutic strategy. Its core design lies in preserving the intact intracellular signaling domains of the CAR construct while replacing the extracellular antigen-binding domain with a specific autoantigen fragment. This fragment enables the selective recognition and elimination of autoreactive B cells expressing pathogenic BCRs, while sparing normal B cells responsible for maintaining humoral immunity (148, 149). This selective targeting mechanism may reduce adverse events such as infections, aligning well with the goals of precision therapy. Its specific mechanism is illustrated in **Figure 3B**. The development of CAAR-T therapy in PMN is supported by the precise identification of antibody-binding epitopes in MN-associated antigens, such as PLA2R1 and THSD7A, with some epitopes refined to single antigenic domains and small molecular weight epitopes (47, 55, 150, 151). Therefore, the fusion of smaller antigen fragments of PLA2R1 or other MN antigens into chimeric receptors appears to be a feasible approach. Furthermore, the N-terminal regions of PLA2R1 and THSD7A constitute immunological hotspots exhibiting the highest reactivity with patient autoantibodies (47, 55, 151). Therefore, CAAR constructs incorporating solely these regions may achieve clearance of the majority of autoreactive B cells, providing critical support for CAAR-T targeting design. Altun et al. successfully designed and developed two chimeric autoantibody receptor (PLA2R-CAAR-T) constructs targeting critical PLA2R epitopes, designated C17 and C178. C17-CAAR-T focuses on three core functional domains of PLA2R: the CysR, CTLD1, and CTLD7. C178-CAAR-T incorporates an additional CTLD8 based on the aforementioned three domains. Research findings indicate that both C17-CAAR-T and C178-CAAR-T effectively bind or neutralize anti-PLA2R autoantibodies. In addition, they significantly reduce the number of pathogenic anti-PLA2R B cells *in vitro*. Extensive analysis and high-throughput membrane proteomics array screening confirmed that neither PLA2R-CAAR-T construct exhibited off-target cytotoxicity, demonstrating favorable safety profiles. Further comparative analysis demonstrated that C17-CAAR-T exhibited slightly superior T-cell surface expression stability, target-specific cytotoxicity, and *in vivo* engraftment persistence compared with C178-CAAR-T, and was therefore designated as the priority candidate for subsequent development (152). Currently, the clinical translation of CAAR-T therapy faces multiple challenges. The epitope spreading commonly observed during the course of PMN may limit the long-term efficacy of single-epitope CAAR-T approaches. In addition, antigen heterogeneity, potential immunogenicity, and the complexity of manufacturing remain major bottlenecks that must be overcome. Future research should focus on the development of multivalent or universal CAAR-T designs to provide safer and more durable precision therapeutic options for patients with PMN.

### 4.3 CAAR-NK cell therapy

NK cells are key effector cells of the innate immune system, playing a central role in regulating immune responses and maintaining immune homeostasis (153, 154). Compared with CAR-T cell therapy, CAAR-NK offers a safer and more accessible antigen-specific cellular therapeutic strategy for PMN. The specific mechanisms by which it eliminates pathogenic cells are illustrated in **Figure 3C**. Engineering CAAR-NK cells that express receptors targeting autoantigens or B-cell–specific receptors offers several advantages: NK cells exhibit low alloreactivity and a significantly reduced risk of graft-versus-host disease compared with effector T cells. Therefore, NK cells do not require strict HLA matching, overcoming a key limitation of allogeneic effector T-cell therapies. In addition, the relatively short lifespan of NK cells helps mitigate the risk of long-term off-target effects, but also necessitates repeated infusions during treatment (155). Furthermore, NK cells can be derived from multiple platforms, including the NK-92 cell line, umbilical cord blood, and induced pluripotent stem cells. This approach circumvents the technical bottleneck of collecting and expanding sufficient autologous T cells in immunocompromised patients, significantly streamlining the production process and reducing treatment costs (155, 156). These characteristics render engineered NK cells a promising candidate for cellular immunotherapy in PMN. As CAAR-NK cells require specific antigen fragments incorporated into their chimeric receptors (148), these fragments for MN can be derived from well-defined pathogenic antigens such as PLA2R or THSD7A. CAARs targeting these antigens can incorporate corresponding immunologically advantageous fragments within their extracellular domains. As pathogenic B cells express BCRs that recognize specific autoantigens, CAAR-NK cells presenting these antigenic fragments can selectively bind to autoreactive B cells. Following synapse formation, CAAR-NK cells release cytotoxic molecules such as perforin and Grz B, ultimately inducing B cell apoptosis (148). Based on this foundation, Seifert et al. constructed a CAAR-NK cell strategy targeting MN pathogenic antigens PLA2R and THSD7A, demonstrating highly specific elimination of autoantibody-producing B cells *in vitro* without affecting non-targeted cells (157). These findings demonstrate that CAAR-NK cell strategy as a precise, antigen-directed cellular immunotherapeutic approach as an alternative or complement to traditional broad immunosuppression in MN. However, CAAR-NK currently remains in the fundamental research stage, and its clinical translation requires further clinical validation.

### 4.4 CAR regulatory T (Treg) cell therapy

The aforementioned chimeric receptor-engineered therapies all focus on eliminating pathogenic cells. In contrast, CAR-Treg combines the precise targeting of chimeric receptors with the immune-regulatory function of Tregs, enabling direct recognition of and localization to regions expressing self-antigens, thereby facilitating highly specific immune modulation in immune regulation (as shown in **Figure 3D**). Regulatory T cell deficiency is commonly observed in patients with PMN (158, 159). Therefore, engineering CAR-Tregs targeting disease-relevant autoantigens represents a promising strategy for suppressing autoimmune responses. Unlike cytotoxic cell therapies, the primary mechanism of CAR-Tregs is to modulate the activation, proliferation, and function of pathogenic lymphocytes, thereby restoring immune homeostasis rather than directly eliminating immune cells. This strategy may reduce infection risks associated with widespread B-cell depletion and demonstrates favorable long-term tolerance (160). Moreover, stable Tregs do not produce proinflammatory cytokines, which may mechanistically lower the risk of CRS associated with conventional CAR-T therapy (161, 162). With advances in technology, recent work by Saleem et al. has further optimized CAR-Treg therapy by developing an inducible CAR-Treg that releases IL-10 only upon CAR–antigen engagement. This design enhances targeted immunoregulation while reducing off-target effects, thereby providing a novel direction for the refinement of CAR-Treg–based therapies (163). Currently, CAR-Treg therapy in the field of kidney transplantation has received clinical trial authorization (NCT04817774) and has achieved preclinical success in other autoimmune diseases (164, 165). Therefore, CAR-Treg therapy is considered to possess high translational potential in the field of PMN.

### 4.5 CAR macrophages (Ms) therapy

**Figure 4:**
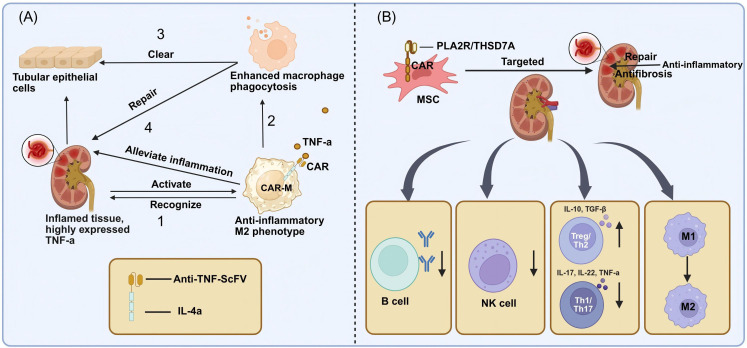
**(A)** Engineered TNF-α–responsive CAR-M selectively target the inflammatory microenvironment in PMN. The synthetic CAR functions as a cytokine-responsive signaling switch that is activated by elevated TNF-α in renal lesions, promoting an anti-inflammatory M2-like phenotype (1). Activated CAR-M exhibit enhanced phagocytic capacity, facilitating the clearance of apoptotic renal cells and contributing to tissue homeostasis (2) (3). In addition, they secrete anti-inflammatory cytokines to attenuate local inflammation and release reparative mediators that support epithelial regeneration and tissue repair (4). **(B)** CAR-MSCs enhance targeted homing and immunomodulatory functions in PMN by recognizing disease-associated antigens (e.g., PLA2R/THSD7A) via their scFv domains. Upon antigen engagement, CAR-MSCs activate intracellular signaling pathways and exert potent immunoregulatory effects. These include suppression of pathogenic B-cell activation and antibody production, attenuation of pro-inflammatory T-cell and NK-cell responses, and modulation of macrophage polarization toward an anti-inflammatory M2 phenotype via paracrine signaling. Concurrently, CAR-MSCs promote Treg differentiation and anti-inflammatory cytokine secretion (e.g., IL-10, TGF-β), ultimately contributing to inflammation resolution, tissue repair, and attenuation of renal fibrosis.

As key effector cells of the innate immune system, macrophages utilize potent phagocytic activity to eliminate pathogens and cellular debris, thereby maintaining tissue homeostasis. In addition, macrophages exhibit regenerative potential and play essential roles in the initiation and resolution of inflammation (166, 167), making them attractive targets for therapeutic intervention. Patients with PMN are typically characterized by a state of chronic inflammation throughout the disease course. Even in early-stage disease, serum levels of inflammatory cytokines, including tumor necrosis factor-α (TNF-α), interferon-γ (IFN-γ), IL-2 and IL-10 are dysregulated in patients with PMN (168), providing a mechanistic rationale for targeting macrophages in PMN. In contrast to CAR-T cells, macrophages exhibit limited *in vivo* expansion capacity compared with CAR-T cells, and the limited cell numbers following their infusion can effectively mitigate the risk of cytokine CRS (169). Furthermore, macrophages exhibit a shorter *in vivo* residence time than CAR-T cells, which further mitigates the potential risk of long-term immune dysregulation (168). In recent years, this therapeutic strategy has been explored in the field of nephrology. A TNF-responsive switch-type CAR-M developed by Cao et al. exhibits three key features: targeted activation, phenotypic stability, and tissue repair capacity. This engineered CAR-M is selectively activated in TNF-high inflammatory microenvironments within the kidney, where it promotes polarization toward an anti-inflammatory (M2-like) phenotype. In parallel, CAR-M enhances phagocytic clearance of apoptotic tubular epithelial cells and facilitates tissue repair (as shown in **Figure 4A**). In models of acute kidney injury and chronic kidney disease, TNF-targeted CAR-Ms therapies have demonstrated significant renoprotective effects (170). This study provides a novel approach for targeted cell therapy of PMN. By constructing engineered CAR-M cells that specifically target key pro-inflammatory mediators in the renal inflammatory microenvironment of PMN, these cells can be precisely activated locally within diseased kidneys and undergo a directed phenotypic switch toward an anti-inflammatory state. This mechanism efficiently suppresses abnormal local inflammatory responses and promotes the repair of damaged renal tissue.

### 4.6 CAR-mesenchymal stem cells (MSCs)

MSCs, owing to their broad immunomodulatory and tissue reparative properties, including suppression of effector T cells (Teff), induction of Treg expansion, and inhibition of B-cell responses, have emerged as important therapeutic candidates for the treatment of autoimmune diseases (171–174). Given the well-defined podocyte target antigens and the dysregulated local glomerular immune microenvironment characteristic of PMN, the CAR-MSC strategy exhibits unique therapeutic potential for restoring renal local immune homeostasis (as shown in **Figure 4B**). To overcome the batch-to-batch variability caused by donor heterogeneity and the limited proliferative lifespan of conventional primary MSCs, immune and matrix regulatory cells (IMRCs) have been developed. IMRCs (immune and matrix-regulating cells) are mesenchymal stem cell–like populations derived from human embryonic stem cells (hESCs). Compared with conventional MSCs, IMRCs exhibit improved batch consistency, enhanced immunomodulatory capacity, prolonged proliferative potential, and anti-fibrotic properties (175). In addition, IMRCs offer practical advantages, including scalable production, robust quality control, and high amenability to genetic modification (175–177). These characteristics lay an important foundation for the further development of CAR-modified engineered cells. Safety assessments have confirmed that IMRCs pose a low risk of pulmonary embolism following intravenous injection and do not induce tumor formation, demonstrating superior safety (175). It is worth emphasizing that IMRCs have demonstrated clear therapeutic efficacy in mouse models of Heymann nephritis. Intravenous infusion of hESC-IMRCs significantly alleviated proteinuria and renal damage in Heymann nephritis *in vivo*. Further mechanistic studies suggest that this effect may be associated with IMRCs upregulating the proportion of Tregs and IL-10 levels, as well as suppressing the expression of pro-inflammatory factors IL-17 and TNF-α (176). Although IMRCs or CAR-IMRCs have not yet entered clinical trials for PMN, this strategy lays the foundation for the development of novel engineered cellular therapies that combine precise targeting with microenvironmental remodeling for PMN. Furthermore, existing studies have confirmed that the route of administration is a key factor influencing the therapeutic efficacy of MSCs (178–180). Therefore, determining the optimal route of IMRC administration for PMN treatment will provide important technical support for the clinical translation of this therapeutic strategy.

## 5 Emerging precision immunotherapy strategies

### 5.1 Targeted protein degradation (TPD) - sweeping antibody technology

**Figure 5:**
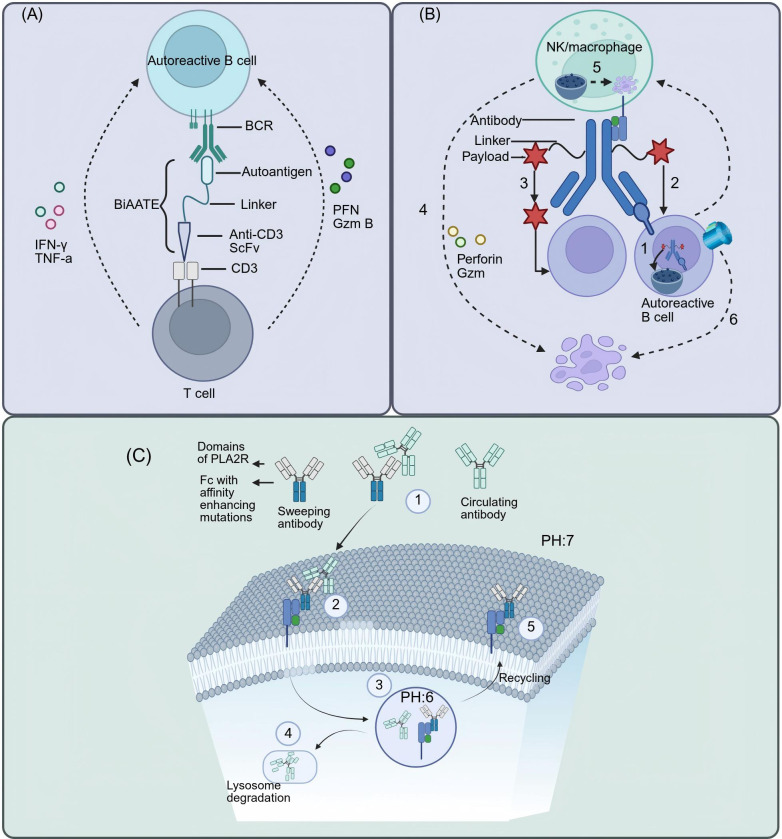
**(A)** BiAATE acts as a bridge, binding to CD3 on the surface of T cells at one end and to BCR on autoreactive B cells at the other, thereby forming an immune synapse, activating T cells and directing cytotoxic T cells to autoreactive B cells.Activated T cells release perforin, granzyme B (GzmB), IFN-γ, and TNF-α to kill autoreactive B cells without affecting normal B cells. **(B)** ADCs consist of three components: an antibody, a linker, and a payload. They eliminate target cells primarily through the following three pathways: 1. Direct internalization-mediated killing: After binding to the target antigen on the cell surface, the ADC antibody forms a complex and is internalized into the target cell. It is then degraded in the lysosome, releasing its payload to exert its killing effect (1). 2. Payload-mediated killing: The payload is released extracellularly, exerting direct cytotoxic effects on target cells (2); simultaneously, it can diffuse to adjacent cells, achieving killing of neighboring cells that do not express the antigen (3). 3. Immune effector-mediated killing: The ADC targets reactive B cells via its Fab fragment, while its Fc fragment recruits effector cells such as NK cells and macrophages and activates the complement system. It mediates target cell apoptosis, phagocytosis, or lysis through three pathways—ADCC (4), antibody-dependent cellular phagocytosis (5), and CDC (6)—thereby eliminating target cells. **(C)** Sweeping antibodies achieve efficient and sustained clearance of circulating soluble antigens through dual engineered modifications: pH-dependent antigen binding and enhanced endocytic uptake and recycling.

Sweeping antibodies are an emerging frontier in antibody engineering developed in recent years based on the concept of TPD, enabling the efficient and selective clearance of circulating pathogenic soluble antigens or immune complexes. This technology relies on antibody engineering to confer pH-dependent antigen-binding properties, allowing stable antigen binding under neutral conditions and rapid dissociation within acidic endosomal compartments (181, 182). Simultaneously, enhanced affinity of the Fc region for FcRn or FcγRIIb is engineered to synergistically accelerate the endocytosis and lysosomal degradation of target antigens through pathways mediated by liver sinusoidal endothelial cells and other cellular mechanisms, while enabling recycling and recirculation of the antibody itself (183, 184). The specific mechanisms by which TPD eliminates pathogenic antigens are illustrated in **Figure 5C**.

With the progressive identification of an increasing number of target antigens, therapeutic sweeping antibodies technology directed against PMN antigens may emerge as a future therapeutic direction. The advantage of this approach lies in the capacity for customized development of distinct sweeping antibodies tailored to target antigens to achieve precise clearance. Previous studies have suggested that the N-terminal domain of PLA2R1 may represent the most promising candidate, with and antigen-binding domains can be engineered to recognize pathogenic epitopes derived from PLA2R1 (47, 150, 185). Following administration, therapeutic sweeping antibodies bind pathogenic autoantibodies in the circulation. The resulting complexes can be endocytosed into cells via FcRn-mediated recycling and FcγRIIb-dependent cellular uptake, leading to lysosomal degradation of pathogenic autoantibodies and enabling recycling of circulating sweeping antibodies. However, this approach has limitations and is not applicable to patients with unknown pathogenic antibodies. Additionally, scavenging antibodies targeting the complement system have been explored. For instance, eculizumab blocks C5 cleavage to prevent membrane attack complex formation, while engineered C5-targeting scavenging antibodies achieve efficient C5 clearance (186, 187). Given the pivotal role of complement activation in MN pathogenesis, sweeping antibody technology targeting complement components also holds broad application prospects. From a therapeutic strategy perspective, sweeping antibodies can be combined with B-cell-targeting therapies, as single targeted agents cannot achieve complete eradication of pathogenic B cells. Sweeping antibody technology reduces pathogenic antibody burden and diminishes immune complex deposition without directly suppressing overall immune system function. Furthermore, current technologies including selective degraders, lysosome-targeting chimera, TRIM-Away, antibody-based targeted chimera, and glue-type targeted chimera also belong to antibody-mediated TPD strategies, represent promising next-generation strategies that merit systematic investigation in PMN (188).

### 5.2 Antibody-drug conjugates (ADCs)

ADCs enable more precise therapeutic intervention by selectively delivering highly potent cytotoxic or immunomodulatory payloads to specific cell populations and are currently being explored for the selective elimination of antibody-secreting cells that drive autoimmune disease progression (189, 190). Its structure and mechanism of action are illustrated in **Figure 5B**. Given the remarkable efficacy of ADCs in depleting aberrant immune clones in rheumatoid arthritis and SLE, this strategy also provides important insights for precision therapy in PMN, with the potential to selectively eliminate antibody-secreting cells without inducing broad systemic immunosuppression (191–194). However, several challenges remain. Currently, no exclusive target exists for PMN. Pathogenic and normal immune cells share high overlap in surface markers, risking off-target effects. Furthermore, pathogenic immune cell populations exhibit substantial heterogeneity and dynamic changes, making single-target ADCs unable to cover all pathogenic subpopulations, with residual pathogenic cells easily causing disease relapse. Long-term administration may also lead to target downregulation, altered endocytic pathways, or adaptive resistance through immune cell subset remodeling. Moreover, unlike tumors, PMN represents a chronic systemic immune response requiring extended treatment cycles, thereby imposing higher stability requirements on linkers. In summary, ADC therapy, with its unique precision treatment advantages, holds broad application prospects in the PMN field. Through targeted antigen screening, optimized molecular design, and rigorous clinical validation, ADCs have the potential to overcome limitations of conventional treatments and emerge as a novel direction for precision therapy in PMN, offering patients a safer and more efficient therapeutic option (191, 194).

### 5.3 Bi-specific AutoAntigen-T cell Engagers (BiAATE) therapy

BiAATEs are antigen-directed bispecific molecules designed with a key domain of pathogenic autoantigen at one end and a scFv targeting the CD3 molecule on T-cell surfaces at the other end, linked by a flexible linker (195, 196). Its structure is shown in **Figure 5A**. The mechanism of action of BiAATEs lies in simultaneously bridging autoreactive B cells expressing specific membrane immunoglobulins with CD3 molecules on T-cell surfaces, thereby forming an immunological synapse between them. This redirects and activates the cytotoxic function of T cells, enabling specific elimination of pathogenic B cell clones while maximally preserving the normal B cell pool (195). In the treatment of PMN, BiAATEs do not rely on broad B-cell markers such as CD20, thereby offering the potential to overcome the limitations of conventional therapies in achieving complete elimination of antibody-secreting cells. Furthermore, its mechanism of action relies on the patients’ own T-cell effector functions, avoiding systemic side effects associated with exogenous cytotoxic agents (195, 197, 198). Preclinical studies provide initial support for the therapeutic potential of BiAATEs in PMN. Animal studies have demonstrated that BiAATEs significantly reduce anti-PLA2R antibody levels in model mice without affecting non-specific B cells. *In vitro* studies have further shown that BiAATEs targeting the PLA2R CysR domain effectively deplete anti-PLA2R B cells derived from MN patients (195). Therefore, this therapy is particularly suitable for high-risk MN patients who are PLA2R antibody-positive, unresponsive to conventional immunosuppression, or experience recurrent relapses. However, BiAATEs therapy remains in the early stages of development. Its safety and efficacy require validation through large-scale clinical trials. Furthermore, animal model studies cannot fully replicate human MN pathology, and the long-term immunological consequences remain unknown. Current research suggests potential risks include CRS resulting from excessive T-cell activation, off-target toxicity affecting normal B cells expressing low levels of target antigens, and the development of ADAs with prolonged use (199). Additionally, its efficacy may be influenced by the functional state of T cells and the immune microenvironment within the patients’ body, potentially limiting its effectiveness in patients with T cell exhaustion or severe immunosuppression. Meanwhile, BiAATEs therapy is only applicable to patients with clearly identified antigens and is ineffective for those who are antigen-negative or have unidentified targets, presenting limitations in its therapeutic scope. However, it is foreseeable that BiAATEs therapy possesses high scalability. Once other MN-associated antigens or pathogenic antigens of other autoimmune diseases are identified, new targeted therapies can be rapidly developed. Deepening understanding of antigen structures like PLA2R will aid in optimizing BiAATEs design, enhancing their affinity and stability.

## 6 Immunomodulatory therapy targeting T cells and cytokines

### 6.1 Therapies targeting Tregs

Patients with PMN commonly exhibit impaired immune tolerance, among which a reduction in Treg number and functional impairment are considered important underlying mechanisms. Specifically, Treg imbalance in PMN is characterized by decreased Treg abundance (200, 201) and diminished activity (159). Treg deficiency may weaken the suppression of autoreactive B cells, thereby driving humoral immune responses against self-antigens. In addition, the CD39^+^ Treg subset has shown unique value in risk stratification and prognostic assessment in PMN, suggesting that restoring Treg-mediated immunosuppressive function may represent a novel therapeutic direction (202).

Current Treg-targeted therapeutic strategies mainly include polyclonal Tregs, antigen-specific Tregs, engineered Tregs, and induced Tregs. Polyclonal Treg cell therapy, particularly autologous polyclonal Treg therapy, demonstrates excellent safety and tolerability while directly enhancing endogenous Treg cell activity. It has shown promising potential in autoimmune diseases such as LN (203, 204), providing important guidance for the application of this therapy in PMN. However, the lack of antigen specificity may limit therapeutic efficiency and require higher cell doses. In contrast, antigen-specific or engineered Tregs offer greater targeting specificity and potentially superior therapeutic efficiency compared with conventional polyclonal Tregs, making them conceptually better suited for PMN, a disease driven by antigen-specific humoral immune responses. However, direct evidence supporting this strategy in PMN remains lacking, and its clinical translation continues to face practical challenges, including antigen selection, cellular stability, and scalable manufacturing. In addition to cell-based approaches, strategies aimed at selectively expanding endogenous Tregs have attracted increasing attention. Among these, low-dose IL-2 therapy represents a particularly promising approach (205). Tregs express high levels of the high-affinity IL-2 receptor and can be preferentially expanded under low IL-2 concentrations that are insufficient to activate conventional effector T cells (206). Importantly, IL-2 signaling can also modulate follicular helper T cell responses and suppress GC activity, thereby indirectly inhibiting B-cell activation and autoantibody production (207). Clinical studies in various autoimmune diseases have demonstrated encouraging efficacy of low-dose IL-2 therapy (208, 209). However, challenges remain, including the lack of standardized dosing regimens and uncertainties regarding long-term safety (208, 210). Overall, Treg-directed therapy offers a strategy for immune tolerance restoration in PMN that is distinct from conventional B-cell depletion approaches. Given that current evidence is still largely derived from other autoimmune diseases, its true efficacy and appropriate target populations in PMN remain to be further validated.

### 6.2 Therapies targeting T helper (Th) 17 cells and related cytokines

An increasing body of evidence suggests that persistent T-cell dysregulation, particularly the activation of Th17-driven inflammatory networks, is an important contributor to refractory disease in patients with PMN. Accumulating clinical evidence indicates that the proportion of Th17 cells and circulating IL-17 levels are significantly elevated in patients with PMN, both in peripheral blood and renal tissue, and positively correlate with disease activity and severity (211, 212). In addition, Th17-driven inflammation has been associated with an increased risk of venous thromboembolism, higher relapse rates, and poorer clinical outcomes (211). These findings indicate that targeting the Th17 axis, either directly or through its regulatory networks, represents a promising complementary therapeutic strategy in PMN.

#### 6.2.1 IL-17A

IL-17A, as a core effector molecule mediating proinflammatory responses in Th17 cells, exhibits abnormally high expression in patients with PMN, closely associated with disease severity and poor prognosis (211, 213, 214). Notably, conventional B-cell–depleting therapy such as RTX has limited effects on Th17-mediated cytokine responses (211), providing a rationale for combination strategies. Current therapeutic approaches include anti-IL-17A monoclonal antibodies (e.g., ixekizumab and secukinumab) and IL-17 receptor antagonists (e.g., brodalumab). Compared with traditional immunosuppressive agents, these targeted therapies offer more selective immunomodulation and may reduce the risk of systemic adverse effects, including infections and bone marrow suppression. However, given the critical role of IL-17 in mucosal barrier defense, blockade of this axis may increase the risk of fungal infections, warranting particularly close monitoring in high-risk patients with PMN (215). Despite their potential, clinical evidence supporting the use of IL-17-targeted therapies in PMN remains limited. Further studies are required to determine their long-term efficacy, optimal patient selection, and safety, particularly in combination with standard therapies.

#### 6.2.2 IL-6

As a key upstream driver of Th17 polarization, IL-6 is also considered a potential contributor to immune dysregulation in PMN. In PMN, IL-6 levels are significantly elevated in both serum and urine, and peripheral blood mononuclear cells exhibit enhanced IL-6 secretion following antigenic stimulation (216–218). Meanwhile, it inhibits Treg cell generation via the STAT3 pathway, thus promoting Th17-mediated renal inflammatory responses. Although IL-6 pathway activation has been implicated in MN pathogenesis, its role appears to be context-dependent. Clinical applications of IL-6 receptor blockade, particularly with tocilizumab, have primarily been reported in cases secondary to idiopathic multicentric castleman disease, where IL-6-driven systemic inflammation is a dominant feature (219, 220). In such settings, IL-6 inhibition has been associated with improvements in proteinuria and inflammatory status. Available IL-6–targeted therapies include anti-IL-6 monoclonal antibodies (e.g., siltuximab and ziltivekimab) and IL-6 receptor antagonists (e.g., tocilizumab). These agents may theoretically suppress Th17 polarization and attenuate inflammatory responses in PMN. However, current evidence suggests that IL-6 may not represent a dominant pathogenic driver in PMN. Experimental studies have shown that IL-6 receptor blockade did not significantly improve proteinuria or histopathological injury in passive Heymann nephritis models (217). Moreover, high-quality clinical data in PMN are lacking, and the therapeutic benefit of IL-6 inhibition appears to be largely confined to specific patient subgroups with concomitant systemic inflammatory conditions. Therefore, further studies are needed to define optimal patient selection and clarify the role of IL-6–targeted therapies in PMN.

#### 6.2.3 IFN-α

IFN-α, as a broad-spectrum antiviral and immunomodulatory cytokine, has been applied in PMN treatment primarily based on its inhibitory effects on Th17 cell differentiation and IL-17 secretion (221). Clinical studies have explored the use of IFN-α in PMN, particularly in hepatitis B virus (HBV)-associated MN. In a cohort of steroid-resistant HBV-associated MN patients, recombinant IFN-α therapy resulted in complete remission of proteinuria within three months, with an acceptable safety profile characterized primarily by mild flu-like symptoms (222). Additional studies have reported improvements in renal outcomes following IFN-α treatment (223). Ongoing clinical trials (e.g., NCT05941845) are further evaluating personalized IFN-α therapy in PLA2R1-positive MN patients. Their findings are expected to provide new evidence-based support for precision therapy of PMN. Despite these promising findings, IFN-α exhibits dual immunological effects. While it can suppress Th17-mediated inflammation and confer renal protection, it also carries the potential to induce or exacerbate autoimmune responses. Consequently, its clinical application requires careful patient selection and risk–benefit assessment. Further large-scale studies are needed to clarify its safety profile and therapeutic role in PMN (224).

### 6.3 Cytokine therapy targeting IL-4 in Th2 cells

IL-4 is a key cytokine regulating B-cell immune responses. In the pathophysiology of PMN, IL-4 plays an important role by promoting B-cell proliferation, differentiation, and immunoglobulin class-switch recombination toward IgG4 (225, 226). In the presence of IL-4, B cells derived from patients with PMN exhibit significantly enhanced IgG4 synthesis and secretion, further supporting its role in promoting pathogenic autoantibody production (226). Given the central role of IL-4 in PMN, targeted therapeutic strategies have been explored. Dupilumab, a monoclonal antibody targeting the IL-4 receptor α (IL-4Rα), inhibits both IL-4 and IL-13 signaling pathways, thereby suppressing Th2-mediated immune responses (227). Two case reports have demonstrated the successful use of dupilumab in patients with refractory membranous glomerulonephritis, achieving remission with favorable safety profiles (228). However, it should be noted that existing studies and case reports suggest that the potent inhibition of Th2-related cytokines by dupilumab may disrupt immune homeostasis, resulting in relative predominance of Th1-type immune responses and potentially compromising immune tolerance. In the field of nephrology, such immune imbalance has been associated with adverse clinical outcomes: in several cases, dupilumab-induced Th1-biased immune responses have not only precipitated anti-GBM nephritis (229), but also triggered *de novo* IgA nephropathy or exacerbation of pre-existing disease (230). Accordingly, when considering dupilumab for MN treatment, comprehensive evaluation of patient immune status, underlying disease conditions, and comorbidities is essential, with vigilant monitoring for exacerbation of renal disease or *de novo* autoimmune nephropathy secondary to immune imbalance to ensure clinical safety.

### 6.4 Targeting T-cell co-stimulatory pathway: CD80/CD86

Cytotoxic T-lymphocyte-associated antigen 4 (CTLA-4) is a critical negative regulator of T-cell activation that functions by outcompeting the co-stimulatory receptor CD28 for binding to B7 molecules (CD80/CD86) on antigen-presenting cells. Through its significantly higher binding affinity, CTLA-4 effectively inhibits CD28-mediated co-stimulatory signaling, thereby attenuating T-cell activation and downstream immune responses (231, 232). Given the central role of CD80/CD86-mediated signaling in GC reactions and immunoglobulin class-switch recombination, modulation of this pathway represents a rational strategy for controlling aberrant humoral immunity. Based on this mechanism, CTLA-4–Ig, a fusion protein consisting of the extracellular domain of CTLA-4 linked to the Fc region of human immunoglobulin, has been developed as an immunomodulatory biologic agent. CTLA-4–Ig suppresses autoreactive T-cell activation and disrupts T cell–B cell interactions, thereby inhibiting autoantibody production. Preclinical studies have demonstrated its efficacy across multiple autoimmune disease models, supporting its potential application in antibody-mediated disorders (233–235). At present, research on CTLA-4-Ig therapy in the field of PMN remains at the preclinical exploratory stage. In animal models of MN, treatment with CTLA-4–Ig significantly reduces proteinuria and serum creatinine levels, ameliorates renal histopathological injury, and suppresses intrarenal Th17 cell activation. Importantly, these effects appear to occur independently of direct reductions in circulating autoantibody levels, suggesting a mechanism driven primarily by modulation of T-cell–mediated immune responses rather than antibody depletion. Interestingly, combination therapy with the plasma cell–targeting agent bortezomib has not demonstrated significant additive therapeutic benefit in experimental MN models (236). This finding implies that CTLA-4–Ig monotherapy may be sufficient to achieve effective immunomodulation in certain contexts, and that targeting upstream T-cell co-stimulatory pathways may exert broader regulatory effects than strategies focused solely on plasma cell depletion. Collectively, these findings highlight the therapeutic potential of targeting the CTLA-4/CD80/CD86 co-stimulatory axis in MN. By modulating T-cell activation and disrupting pathogenic T cell–B cell interactions, CTLA-4–Ig provides a mechanistically distinct approach that complements existing B-cell–directed therapies. Further studies are warranted to evaluate its clinical efficacy, optimal therapeutic positioning, and potential role in combination treatment strategies for PMN.

## 7 Complement inhibitors

**Figure 6:**
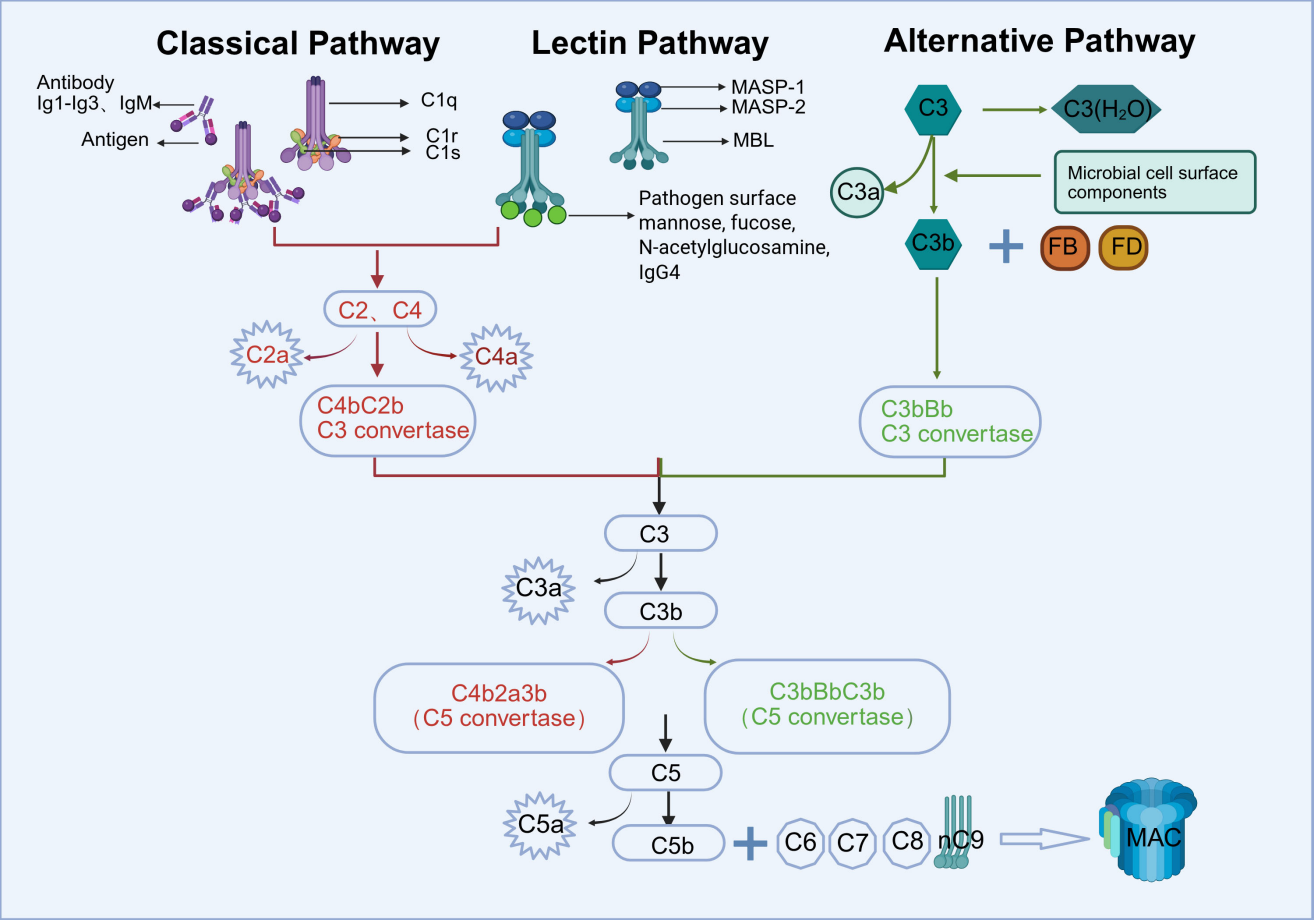
The complement cascade can be activated through three distinct pathways: the classical pathway (CP), the lectin pathway (LP), and the (AP). These pathways cross-regulate each other and converge at the C3 convertase, entering a common terminal pathway that ultimately assembles into the membrane attack complex (MAC). The MAC forms transmembrane pores in the target cell membrane, directly mediating cell lysis. Complement activation also generates multiple soluble effector molecules. Among these, anaphylatoxins C3a, C4a, and C5a recruit and activate immune cells, serving as key mediators of complement-associated inflammatory responses.

Immunofluorescence microscopy in patients with PMN reveals deposits of IgG and complement components, suggesting a central role of the complement system in the pathogenesis of MN. The complement activation pathways are illustrated in **Figure 6**. Extensive research has indicated that C3 and complement component 5b-9 (C5b-9) are detectable in the glomeruli of nearly all types of MN patients (237–239). These findings imply that terminal pathway-mediated podocyte injury represents a common pathological outcome regardless of the initial complement activation pathway. Currently, there is no consensus on whether complement is activated primarily through the classical, lectin, or AP in MN. In PMN, complement activation is not a fixed linear process but rather a highly heterogeneous and dynamic network, posing challenges for precision targeting in treatment-resistant patients. The mode of complement initiation depends on the characteristics of the target antigen, the composition of antibody subclasses, and their Fc glycosylation profiles. For example, in cases where antigens are predominantly targeted by IgG4, such as PLA2R1, THSD7A, high temperature requirement a serine peptidase 1, and CNTN1, LP activation may predominate (240–242). In contrast, membranous nephropathy associated with EXT1/EXT2, typically characterized by IgG1-dominant responses, is more likely to involve activation of the CP (243, 244). Importantly, the pathogenic role of IgG4 in PMN is closely linked to Fc glycosylation. Aberrant glycosylation patterns, including altered galactosylation and enhanced mannose-binding lectin recognition, may facilitate LP activation despite the intrinsically low complement-binding capacity of IgG4. These findings underscore the importance of antibody structure, in addition to subclass, in determining complement activation pathways (245). Furthermore, the heterogeneity of complement activation exhibits distinct temporal characteristics. Comparative studies of PMN at different stages have revealed that IgG1 predominates in the early phase, whereas IgG4 becomes the dominant isotype in later stages (246). This suggests that IgG subclass switching may occur in the antibody response targeting PLA2R. This dynamic IgG subclass switching essentially reflects class switching and gene rearrangement during the maturation of B-cell immune responses, implying that the same patient may experience different complement initiation patterns at different disease stages (247). Regardless of the initial trigger, the AP primarily functions as an amplification loop, reinforcing complement activation and driving convergence toward terminal pathway activation (248).

The complement inhibitors currently under investigation in the MN field are summarized in the **Table 1** (218, 249–256). Complement inhibitors act primarily as downstream effector blockers, preventing complement-mediated tissue injury without directly suppressing upstream immune activation. As such, complement inhibition alone may be insufficient to halt the formation of immune complexes driven by ongoing B-cell activity. Therefore, complement-targeted therapies may benefit from combination with B-cell–directed or immunomodulatory strategies to simultaneously suppress autoantibody production and downstream effector mechanisms. In addition, blockade of a single complement activation pathway may lead to compensatory activation of alternative pathways (257). Therefore, when applying complement inhibitors, selection of upstream complement inhibitors combined with amplification loop inhibitors (factor B/D inhibitors) may be considered to simultaneously restrict initiation signals and block the self-amplification of the complement cascade. Recent advances have led to the development of next-generation complement-targeted therapies aimed at improving both efficacy and safety through precision modulation. One promising approach involves enhancing endogenous complement regulation at the cellular level. Activation of the melanocortin 5 receptor signaling pathway in podocytes has been shown to downregulate factor B and factor D expression via peroxisome proliferator-activated receptor gamma-dependent mechanisms. This strategy suppresses the complement amplification loop intrinsically and significantly attenuates glomerular injury in experimental MN models (258). Another innovative strategy is tissue-directed complement inhibition. The C3d-targeted antibody fusion protein C3d-mAb-2fH (and its humanized derivative ADX-097) selectively delivers factor H regulatory domains to sites of complement activation. By targeting C3d-enriched regions, this approach enables localized inhibition of the AP while preserving systemic complement function (259). These precision-targeted strategies represent a paradigm shift from systemic complement blockade toward site-specific and pathway-selective modulation, with the potential to enhance therapeutic efficacy while minimizing adverse effects. These next-generation strategies hold promise for significantly improving safety while enhancing therapeutic efficacy, laying a solid foundation for the clinical translation and precision application of complement inhibitors.

**Table 1:** Research progress of complement inhibitors in PMN.

Complement hierarchy	Primary target	Representative drugs	Evidence phase	Mechanism	Advantages	Limitations
LP	MASP-2	Narsoplimab	Phase II Clinical Trial (NCT02682407).	By binding to C4 substrate, it competitively inhibits the protease activity of MASP-2, thereby blocking C4 cleavage and suppressing the initiation of the complement activation pathway (249).	Selectively inhibiting the complement pathway while preserving immune surveillance functions of the classical and alternative pathways, with relatively good safety.	This treatment is only effective for the LP and may have limited efficacy for PMN patients primarily affected by the bypass pathway.
Alternative Approach	Factor B	Iptacopan	Phase II Clinical Trial Termination(NCT04154787).	By binding to C3b, inhibitory factor B blocks the formation of the C3 convertase, thereby suppressing amplification in the AP (250).	Blocking the bypass pathway while preserving the upstream defense functions of the CP and the complement pathway.	Interim analysis did not demonstrate superiority over RTX in improving proteinuria, and efficacy has not yet been established.
	Factor D	BCX9930	Phase II Clinical Trial Termination (NCT05162066).	Selectively inhibit factor D activity, block the formation of C3 convertase in the alternative pathway, and suppress AP initiation and amplification (251).	High target specificity and strong blocking efficiency.	Clinical trial terminated early due to insufficient clinical data.
C3	C3/C3b	Pegcetacoplan	The primary endpoint of Phase II was not met (NCT03453619).	Binds to C3/C3b, inhibits C3 convertase activity, and blocks downstream cascade reactions in all three complement pathways (218).	Blocking all three complement pathways provides more comprehensive inhibition of complement activation in PMN.	Due to its position downstream of complement activation, the risk of infection is high; clinical efficacy has not yet been confirmed.
Allergen	C3aR	SB290157 JR14a	Primarily for animal and *in vitro* studies.	Blocking the binding of C3a to C3aR inhibits inflammatory responses in podocytes.	The C3a/C3aR pathway is a key mechanism for complement-mediated podocyte injury; inhibiting this pathway reduces podocyte damage (252).	Lacking clinical evidence, most remain at the experimental research stage.
	C5aR	Avacopan	In other renal diseases such as C3 glomerulopathy and ANCA-associated glomerulonephritis (253).	Antagonize C5aR to inhibit C5a-mediated neutrophil activation and inflammatory amplification (254).	Low risk of bacterial infection in coated capsules (254).	No systematic clinical studies have been conducted in PMN.
C5b-9	C5	Eculizumab	Small-Sample Exploration.	Monoclonal antibodies bind to C5, blocking its cleavage into C5a and C5b, thereby inhibiting C5b-9 complex formation (253).	Directly blocks damage to podocytes.	C3a may independently cause disease without affecting upstream C3a production (255).
		Ravulizumab	Other Clinical Studies on Kidney Diseases (NCT04564339).	Long-acting C5 inhibitor, blocking terminal complement activation (256).		

## 8 Others

In addition to immunosuppressive therapies, extracorporeal therapeutic strategies such as therapeutic plasma exchange and immunoadsorption have been explored as adjunctive options in rituximab-resistant PMN. These approaches aim to rapidly remove circulating autoantibodies and immune complexes, thereby alleviating complement activation and podocyte injury. Clinical observations suggest that such interventions may provide short-term benefits, particularly in patients with high autoantibody titers or severe nephrotic syndrome (181, 260–265). However, as these strategies do not target the underlying autoreactive B-cell population, antibody re-synthesis frequently occurs, limiting their long-term efficacy. Therefore, plasma separation techniques are more commonly considered as bridging or combination therapies rather than definitive treatment options. A comparative overview of different plasma separation techniques, including their separation characteristics, advantages, and limitations, is summarized in **Table 2**.

**Table 2:** Comparison of therapeutic plasma separation technologies.

Technical name	Separation characteristics	Advantages	Limitations
Plasma exchange	Non-selective, whole blood plasma separation and discard.	Broad-spectrum, fast-acting.	High costs and significant infection risks.
Double filtration plasmapheresis	Semi-selective, targeted removal of macromolecular components.	High antibody clearance efficiency and excellent safety profile.	High precision requirements for equipment and filter membranes.
Immunoadsorption	Highly selective, specifically binds to pathogenic substances.	Highly targeted, high safety profile, no exogenous replacement fluid required.	Adsorption columns are costly and cannot suppress antibody production.
Semi-specific immunoadsorption	Class selectivity, preferential binding to IgG subclasses.	High safety, no replacement fluid required.	Moderate antibody clearance efficiency.

Emerging evidence has highlighted a potential role of the gut–kidney axis in renal diseases, providing a theoretical basis for microbiota-targeted therapeutic strategies. Dysbiosis of the gut microbiota has been associated with systemic immune dysregulation, including alterations in T-cell subsets and enhanced humoral immune responses (211, 266–269). Modulation of the gut microbiome through fecal microbiota transplantation may contribute to restoring immune homeostasis and attenuating autoimmune activity in patients with MN (270, 271). Although preliminary findings are encouraging, current evidence remains limited, and the mechanistic links between microbiota alterations and MN progression require further elucidation. Large-scale clinical studies are needed to evaluate the therapeutic efficacy and safety of microbiome-based interventions in MN.

## 9 Conclusions

This review systematically summarizes the major mechanistic basis of RTX-resistant MN and the corresponding therapeutic advances, highlighting a transition in the field from conventional B-cell depletion toward a new stage of precision intervention. Current therapeutic exploration has expanded beyond direct B-cell targeting to encompass multiple key pathogenic processes, including B-cell survival and differentiation, aberrant T–B cell interactions, and complement activation, while further extending to engineered cell therapies, antibody-based bioengineering technologies, and emerging immunomodulatory strategies. Collectively, these advances suggest that the therapeutic paradigm for RTX-resistant MN is shifting from single-target replacement toward coordinated remodeling of multiple pathogenic pathways. Nevertheless, most emerging therapies remain at the preclinical or early clinical stage, and their long-term efficacy, safety profile, target populations, and translational feasibility require further validation. Looking ahead, with the continued expansion of pathogenic antigen discovery, deeper insights from single-cell and spatial omics, and the rapid development of gene editing and targeted delivery technologies, the treatment of RTX-resistant MN is expected to move beyond empirical immunosuppression toward truly mechanism-driven precision immune reprogramming. Establishing a stratified management framework tailored to distinct resistance mechanisms, together with promoting the clinical translation of multi-pathway synergistic interventions, may represent the key direction for future breakthroughs in refractory PMN.
